# Automated citation searching in systematic review production: A simulation study

**DOI:** 10.1017/rsm.2024.15

**Published:** 2025-03-07

**Authors:** Darren Rajit, Lan Du, Helena Teede, Joanne Enticott

**Affiliations:** 1 Monash Centre for Health Research and Implementation, Faculty of Medicine, Nursing, and Health Sciences, Monash University, Clayton, Victoria, Australia; 2 Department of Data Science and AI, Faculty of Information Technology, Monash University, Clayton, Victoria, Australia; 3 Monash Partners Academic Health Sciences Centre, Clayton, Victoria, Australia

**Keywords:** automation, evidence synthesis, guideline development, learning health systems, scoping review, systematic reviews

## Abstract

Bibliographic aggregators like OpenAlex and Semantic Scholar offer scope for automated citation searching within systematic review production, promising increased efficiency. This study aimed to evaluate the performance of automated citation searching compared to standard search strategies and examine factors that influence performance. Automated citation searching was simulated on 27 systematic reviews across the OpenAlex and Semantic Scholar databases, across three study areas (health, environmental management and social policy). Performance, measured by recall (proportion of relevant articles identified), precision (proportion of relevant articles identified from all articles identified), and F1–F3 scores (weighted average of recall and precision), was compared to the performance of search strategies originally employed by each systematic review. The associations between systematic review study area, number of included articles, number of seed articles, seed article type, study type inclusion criteria, API choice, and performance was analyzed. Automated citation searching outperformed the reference standard in terms of precision (p < 0.05) and F1 score (p < 0.05) but failed to outperform in terms of recall (p < 0.05) and F3 score (p < 0.05). Study area influenced the performance of automated citation searching, with performance being higher within the field of environmental management compared to social policy. Automated citation searching is best used as a supplementary search strategy in systematic review production where recall is more important that precision, due to inferior recall and F3 score. However, observed outperformance in terms of F1 score and precision suggests that automated citation searching could be helpful in contexts where precision is as important as recall.

## Highlights

### What is already known?

- Citation searching has been recommended as s supplementary search method in systematic review production; however, manual methods are expensive in terms of effort and time.
- The rise of bibliographic aggregators such as OpenAlex and Semantic Scholar presents promise for automated forms of the technique, but there have been limited studies as to how they perform against standard search methods, what factors may influence performance, and how best to integrate this into existing systematic review production workflows.

### What is new?

- Our work simulated automated citation searching across 27 systematic reviews across three different study areas (biomedical sciences/health, social policy, and environmental management) and two different bibliographic aggregators (OpenAlex and Semantic Scholar).
- This work is novel as citation searching is often recommended as a supplementary method; however, there is limited empirical evidence evaluating automated forms of the techniques, particularly across disciplines, and over different databases.
- The method outperformed standard search strategies in terms of efficiently retrieving articles that are relevant to a systematic review question efficiently (measured by precision) but was not effective in retrieving all possible relevant articles as a whole (measured by recall).
- Study area was found to significant influence performance, with performance being higher in the environmental management literature, compared to the social policy literature.

### Potential impact for *Research Synthesis Methods* readers

- We found that automated citation searching that leverages direct citations should be used as a supplementary search strategy, rather than a stand-alone strategy.
- However, due to its better efficiency, it can be integrated without overly burdening downstream workload in terms of title and abstract screening.
- Teams who wish to integrate the technique should consider the citation activity of authors in their area, as the technique may perform better in areas where “signpost” articles are common. For example, articles such as consensus statements, guidelines or diagnostic criteria.

## 1 Introduction

Systematic review production and associated forms of evidence synthesis are crucial toward ensuring the best external evidence is used to inform policy and clinical practice. However, while traditional systematic review methods are robust, they are mainly manual. This presents a mismatch between the time it takes to synthesize and translate research evidence and the pace of research evidence production.^1^ Given that efficient evidence synthesis is key to “learning health systems,” where evidence from stakeholders, research, practice, and implementation is seamlessly integrated to drive healthcare improvement, new methods for evidence synthesis are needed to improve efficiencies, while maintaining requisite rigor.^1^

In response, the past decade has seen the rise of technological enablers to support evidence synthesis. Mega bibliographic databases such as OpenAlex^2^ and Semantic Scholar^3^ now provide programmatic access via application programming interfaces (APIs) to aggregate subject specific sources such as PubMed and preprint servers such as ArXiV and MedRXiv. This enables potential automation of evidence retrieval that utilizes citations networks and links as sources for articles that are potentially relevant for a particular review question.

Specifically, citation searching or “snowballing” leverages citations and references (citation network) of a “seed” article, for retrieving relevant articles for a particular systematic review question.^4^^–^ ^6^ This relies on the citation activity of article authors and the implicit knowledge contained in these citation links to identify relevant articles. This has advantages over the de facto standard, the Boolean-logic based keyword search, due to not needing to rely on the systematic review team’s own knowledge of potential keywords thus potentially improving the comprehensiveness of a search strategy, particularly in instances where terminology is not well defined.^5^ However, employing citation searching as a supplementary search strategy in conjunction with Boolean-logic-based keyword searches is slow when conducted manually. The adoption of automated methods that leverage APIs such as OpenAlex and Semantic Scholar offers substantial efficiencies in evidence retrieval phases of systematic reviews. This is particularly in living guidelines^7^ and maps.^8^ A recent scoping review^5^ uncovered two examples of such tools: CitationChaser, an opensource R application that leverages the Lens.org database,^9^ and CitationCloud, a publicly available extension of PubMed that allows the visualization of the citation network of an individual paper, with a focus on biomedical sciences.^10^

However, there has been limited investigation into how automated citation searching performs when compared against current standard methods. There is limited guidance on how automated citation searching may be integrated into systematic review workflows, and on optimal circumstances for the technique. Additionally, given the reliance of the technique on data availability and citation activity across different study areas, understanding of potential biases and limitations is crucial.

## 2 Aims

The study aims to:

1. Simulate and evaluate the use of exclusively automated citation searching for evidence retrieval compared to reference standard search strategies employed in systematic reviews. We will examine this approach across three broad study areas: Public health and biomedical sciences; environmental management; and social policy.
2. Evaluate the factors that influence the performance of automated citation searching, including i) automated citation search parameters, ii) review question and included article parameters, and iii) seed article parameters.

## 3 Methods

**Figure 1:**
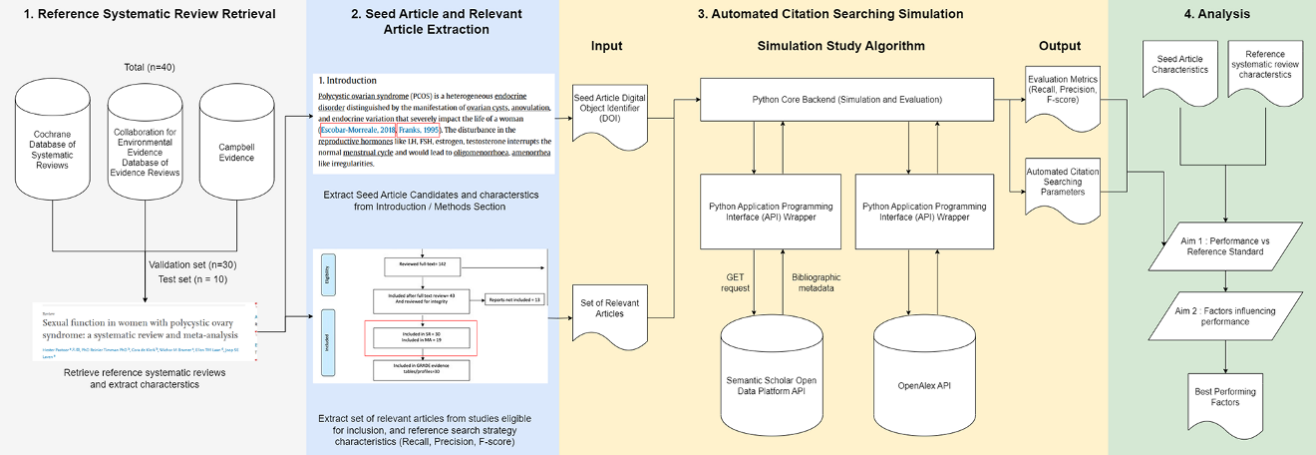
Framework depicting high level methodology of the simulation study. Adapted from protocol (11).

A protocol has been published *a priori.* ^11^ Figure 1 highlights the high-level approach repeated in sample systematic reviews. Python code and data devised to run the simulation and subsequent analyses is available on GitHub (https://github.com/darrenkjr/automated_citation_search_study).

### 3.1 Reference systematic review retrieval

Systematic reviews were randomly selected as outlined in the protocol^11^ and screened against prespecified criteria outlined in Table 1 for inclusion in the dataset. Ten systematic reviews from each of the three study areas were randomly selected producing a random sample of 30 to be screen using the inclusion and exclusion criteria (Table 1). The three study areas and relevant databases were: Public health and biomedical sciences captured through the *Cochrane Database of Systematic Reviews (CSDR)*; environmental management captured through the *Collaboration for Environmental Evidence Database of Evidence Reviews (CEEDER)*; and social policy captured through *Campbell Reviews*.

**Table 1:** Inclusion and exclusion criteria for sample systematic reviews included in study^11^

Inclusion criteria	Exclusion criteria
Completed systematic reviews with available data on search strategy depicted by a PRISMA/Study Flow diagram and included articles. Completed from 2019 to 2022 in English	Systematic reviews with no data on Boolean-based search strategy (including number of included and excluded articles), and no details on included articles

Key characteristics (Title, Source Database, Publication Year, Search Strategy Type, Study Type Inclusion Criteria, Peer reviewed literature vs Grey Literature vs Peer reviewed & Grey Literature, Number of Included Articles) of each systematic review were also retrieved (Supplementary Tables S1 and S2).

### 3.2 Included article extraction

All articles originally deemed eligible for inclusion by original systematic review authors in the data extraction phase of reference systematic reviews were extracted. These are denoted as “Included articles” in the rest of this paper. Where possible, the titles, abstracts, and relevant unique identifier (Digital Object Identifier (DOI), PubMed Identifier (PMID), or Microsoft Academic Graph Identifier (MAG ID)) were also retrieved Included articles were then used to both i) compute the intracluster semantic similarity of each corresponding reference systematic review and as a ii) reference standard to evaluate the performance of the original search strategies. Detailed original search strategies are available in the Supplementary Appendix (Table S1).

### 3.3 Intracluster semantic similarity calculations

Titles and abstracts of included articles were used to compute the intracluster semantic similarity of each corresponding reference systematic review. This represented thematic coherence or topic complexity for each review.

First, the titles and abstracts were encoded as numerical vectors, known as embeddings. The semantic similarity between included articles for each systematic review was calculated using cosine similarity, where the cosine of the angle between two vectors (encoding the representation of the title and abstract of a particular included article) is measured. A cosine value would range from −1 to 1, where −1 would imply opposite meanings, 0 would imply no similarity at all between texts, and 1 indicating identical content.

The pairwise cosine similarity between the vectors of all included articles’ combined titles and abstracts was then computed for each systematic review. The intracluster semantic similarity for a particular reference systematic review would then be determined by averaging these pairwise cosine similarity scores. A higher intracluster semantic similarity would thus suggest a more focused and specific systematic review topic, while a lower score would imply a broader or more complex systematic review topic.

### 3.4 Reference search strategy performance

Included articles were used as the reference standard or “ground truth” for evaluating the performance of the original search strategy of each reference systematic review. Recall, precision, F1 score, F2 score, and F3 score were employed as performance measures (Table 2).

**Table 2:** Performance measures employed in study (recall, precision, and F score)

Measure	Description	Formula^a^
Recall	% Of included articles that were retrieved by method (*Each systematic review has on Y included articles. Recall is the % of these articles that our citation searching method identifies*).	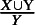
Precision	% Of included articles that were retrieved among all retrieved articles by method (*Precision is % of the systematic review articles identified from all the articles our citation searching method identifies).*	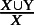
F- 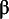 score, 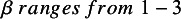	Weighted harmonic mean of both recall and precision, where the 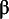 parameter determines the weight of recall in the score. The 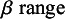 is set at 1–3 to evaluate the performance of citation mining over a range of potential applications (*F scores put more weight towards recall than precision, and vice versa, by different degrees*).	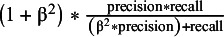

a *X* is the total retrieved documents obtained from a particular search strategy (original search strategy employed by reference systematic review or automated citation searching), and *Y* is the total set of relevant documents eligible for inclusion in the sample systematic review.

To compute recall for the reference search strategy of each reference systematic review, it was assumed that all relevant eligible articles were retrieved (Y in Table 2). Thus, recall was set at 100% for all reference search strategies. To compute precision, the number of all articles retrieved per systematic review (X in Table 2) was extracted from the systematic review PRISMA diagram or results section, and the formula as in Table 2 applied. Consequently, F- 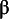 scores were computed assuming 100% recall in the case of the reference search strategies employed by each reference systematic review and applying the formulae in Table 2.

### 3.5 Seed article extraction

In order to simulate the worst-case scenario in which systematic reviewers have no prior knowledge of the current state of the literature, it is assumed that systematic reviewers will select articles that both i) represent their review question at hand and ii) would presumably be cited by authors of articles that should be included in systematic review. For example, articles that represent underlying consensus in a study area such as prior reviews, consensus definitions, or outcome constructs. It is further assumed that such articles are typically cited in the background section to justify the conducting of the systematic review (e.g., needing to update a prior review), or in the methods section as a way to specify the inclusion or exclusion criteria (e.g., citing a consensus definition of a chronic disease to define the population component of a PICO question). Thus, articles from these sections were extracted as seed articles, forming a corresponding seed article pool for each sample systematic review.

The i) DOI/PMID, ii) Title, iii) year published, iv) number of citations, and v) number of references were then retrieved. Seed articles were classified as: Research Article, Evidence Syntheses, Consensus Article, Methodology Article, Commentary Article, Framework Article, and Other (including grey literature such as book chapters and reports).

Articles that i) were included articles but also cited in the background or methods section, or ii) had more than 10,0000 citations, or iii) did not have a retrievable DOI or PMID were excluded from the seed article pool. Included articles were excluded due to potentially introducing bias as the simulation study is meant to simulate the worst-case scenario where teams have no prior knowledge, and articles more than 10,000 citations were excluded due to practical considerations and computational limitations (See Supplementary file for details).

### 3.6 Automated citation searching simulation

Seed articles were used to kickstart automated citation searching processes for each corresponding systematic review. This was conducted on two database APIs: OpenAlex and Semantic Scholar. Both were chosen due to their extensive coverage of over 200 million records that incorporates Microsoft Academic Graph,^12^^,^ ^13^ which has been shown to have superior coverage over database alternatives such as Dimensions, Scopus, and CrossRef.^14^ Further, API access to both databases was provided free of charge for research purposes.

**Figure 2:**
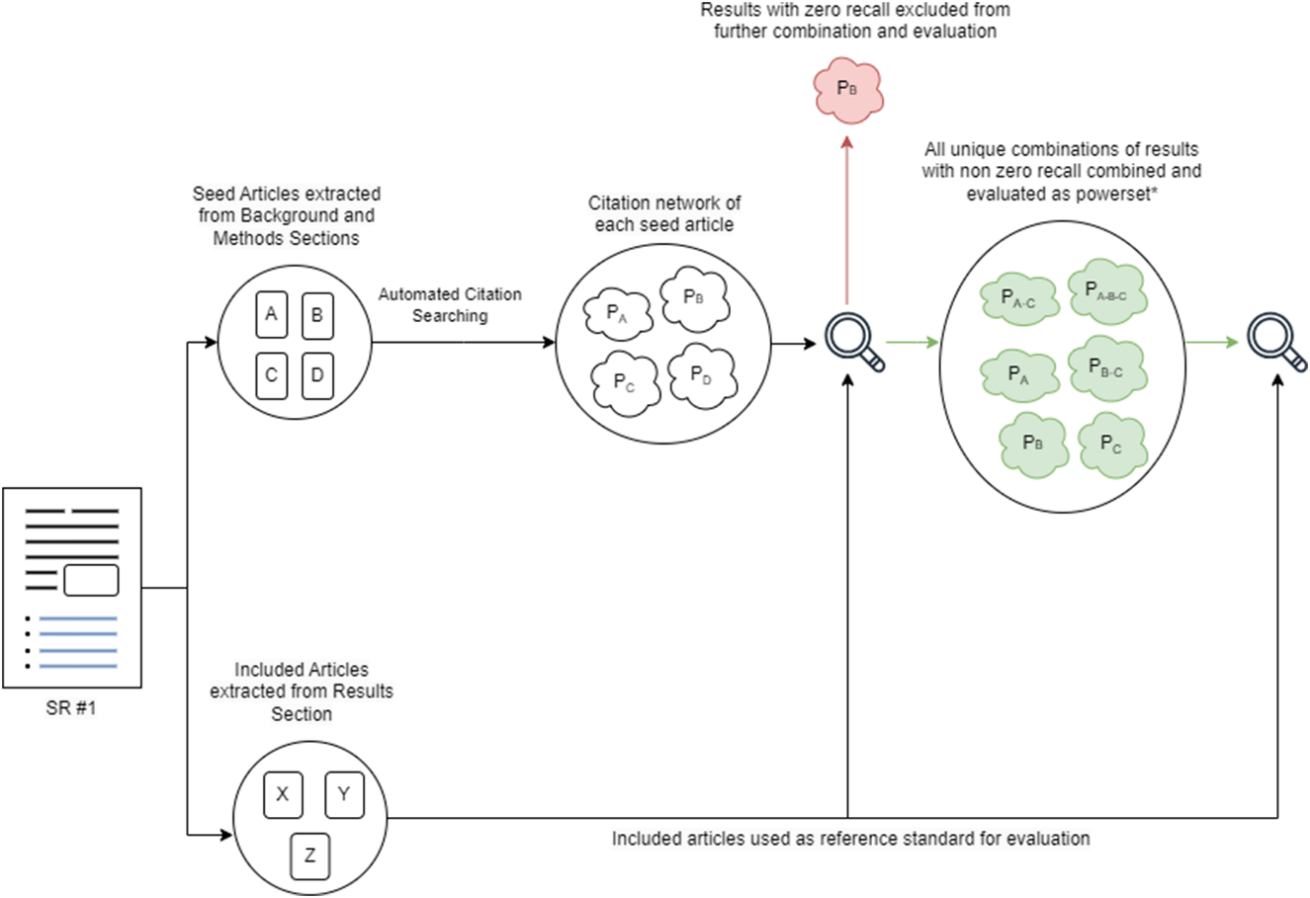
Schematic depicting the automated citation searching simulation process.

As in Figure 2, automated citation searching yielded a citation network for each seed article. Only direct citations (both backward and forward) that were within one hop of the citation network for a specific seed article were retrieved. This citation network was then evaluated according to recall, precision and F1–F3 score, utilizing the included articles as the reference standard for evaluation, and applying the formulae in Table 2.

Results that had a recall of 0 were excluded from further evaluation. All possible unique combinations of the citation networks of each seed article were then iteratively combined and evaluated. Each unique combination that was evaluated at this stage was recorded as an individual citation searching run. Due to computational constraints, only the citation networks of the top 10 seed articles in terms of recall per systematic review were combined and evaluated. In situations where there were less than 10 seed articles with non-zero recall, all seed article citation network combinations that had a non-zero recall were evaluated.

### 3.7 Analysis

The top-performing automated citation searching run for each sample systematic review were then identified based on recall (with F3 score as a tiebreaker) and compared to performance of the reference search strategy of the systematic review, irrespective of the API used.

Three main categories of factors were examined in relation to automated citation searching performance, as quantified by recall, precision, F1 score, F2 score, and F3 score. First, factors related to the review question: intracluster semantic similarity, study type inclusion criteria (gray literature vs peer-reviewed literature), and study area (*CEEDER* vs *Campbell* vs *Cochrane*). Second, seed article characteristics, specifically study type and third, citation searching parameters, including API choice (OpenAlex vs Semantic Scholar) and the number of seed articles used were examined. The spearman’s rank correlation matrix was employed for all numerical variables, whereas the Kruskal–Wallis and post-hoc Mann–Whitney *U* hypothesis test was employed for categorical data (study area, study type inclusion criteria, seed article study type, and API choice). Both approaches were chosen due to the presence of outliers. Analysis for each factor except for “API Choice” was conducted on the top performing run for each systematic review, irrespective of the API used. For the factor of “API choice,” the best performing run for each systematic review generated from each individual API was extracted and compared.

### 3.8 Extensions from initial protocol

In execution, some protocol modifications were required (11). See Supplementary File for details.

## 4 Results

### 4.1 Dataset

Systematic reviews were randomly selected (*n* = 30) and screened against prespecified criteria outlined in Table 1 for inclusion in the dataset. Of the originally planned sample size of 30, only 27 systematic reviews met inclusion and exclusion criteria (Table 1), 10 from *CEEDER*, 9 from *Campbell Systematic Reviews*, and 8 from *CDSR*.

The dataset is composed of 27 systematic reviews, consisting of 10 systematic reviews from the *CEEDER*, representing the environmental management literature, 9 from *Campbell Reviews* representing the social policy literature, and 8 from the *CDSR*, representing the health literature.

In total, 25.9% (7/27) of systematic reviews included only peer-reviewed literature as part of their inclusion criteria, while 74.1% (20/27) included both the grey and peer-reviewed literature. Reviews from the *CSDR* were most likely to only consider peer-reviewed literature (7 out of 8), whereas only 1 out of 10 reviews from *CEEDER* considered peer reviewed literature only. Lastly, all *Campbell Reviews* considered both peer-reviewed and gray literature.

All systematic reviews employed Boolean search strategies. The most common supplementary search strategy was handsearching of specific journals and repositories (59.3%, *n* = 16) followed by backward citation searching only (48.1%, *n* = 13), expert consultation (40.7%, *n* = 11), a full citation search of select articles (33.3% *n* = 9), screening articles from previous versions of the review (11.1% *n* = 3), screening articles from a prior evidence map (11.1% *n* = 3), crowdsourcing through social media (7.4%, *n* = 2), and forward citation searching only (3.7%, *n* = 1).

As in Table 3, each systematic review contained a median of 42 (interquartile range [IQR]: 51.5) eligible articles (included article). Review topic complexity as measured by intracluster semantic similarity was moderate, with an average of 0.846 (IQR: 0.065).

**Table 3:** Median number of included articles (IQR) and average intracluster semantic similarity (±SD) for systematic reviews in each source database, and all reviews in the dataset

Source database	Number of included articles (IQR)	Intra-cluster similarity ±SD)
CEEDER	50.5 (28.25)	0.830 (0.078)
Campbell	22.0 (53.0)	0.829 (0.035)
CDSR	15.0 (58.0)	0.864 (0.047)
All reviews	42.0 (51.5)	0.846 (0.065)

No significant differences in the number of included articles and intracluster semantic similarity were observed across the study areas.

A median of 29 (IQR: 31) seed articles were extracted from each systematic review, resulting in a total of 1024 seed articles extracted. This consisted of 35.7% (*n* = 366) research articles, 26.4% (*n* = 270) evidence synthesis articles, 17.7% (*n* = 181) methodology articles, 11.4% (*n* = 117) commentary articles, 2.44% (*n* = 25) framework articles, and 1.56% (*n* = 16) consensus articles. An additional 4.76% (*n* = 49) articles were classified as “Other,” composed of the gray literature.

Table 4 depicts baseline characteristics of the seed articles retrieved from OpenAlex and Semantic Scholar respectively. Overall, the median number of references per seed article was higher in the Semantic Scholar compared to the OpenAlex API. This was similarly the case for both median citations per seed article and median citation network size per seed article.

**Table 4:** Summary baseline characteristics of seed articles successfully retrieved from the OpenAlex and semantic scholar APIs

				Median citation
		Median references	Median citations	network size
Article type	Article count	(IQR)	(IQR)	(IQR)^a^
*Semantic scholar API*				
Overall^b^	984	47.0 (53.0)	123.5 (348.25)	189.0 (393.25)
Consensus article	14	28.5 (37.25)	360.5 (1187.5)	476.5 (1193.75)
Methodology	174	42.0 (41.25)	457.0 (2650.25)	490.5 (2902.0)
Commentary	114	53.0 (67.75)	176.0 (542.5)	247.5 (521.75)
Evidence synthesis	261	79.0 (67.5)	107.0 (217.5)	205.0 (268.0)
Framework	23	60.0 (54.0)	138.0 (298.5)	216.0 (268.0)
Research article	353	39.0 (34.5)	89.0 (163.5)	136.0 (169.5)
Other^c^	45	3.0 (42.0)	116.0 (486.0)	174.0 (632.0)
*OpenAlex API*				
Overall^b^	1010	30.0 (35.75)	95.0 (277.75)	140.0 (309.5)
Consensus article	15	21.0 (17.5)	623.0 (1327.5)	630.0 (1332.5)
Methodology	179	27.0 (28.0)	372.0 (2314.0)	396.0 (2307.5)
Commentary	117	24.0 (37.0)	140.0 (353.0)	192.0 (369.0)
Evidence synthesis	268	51.5 (49.5)	80.5 (166.5)	151.0 (213.5)
Framework	24	36.5 (36.0)	93.5 (152.0)	140.0 (163.25)
Research article	360	25.0 (25.0)	74.0 (141.25)	106.5 (148.0)
Other^c^	47	0.0 (8.5)	34.0 (138.0)	45.0 (154.0)

a Represents sum of number of references and citations.

b Aggregate of all article types. Only candidates with retrievable DOIs were extracted and retrieved.

c Grey literature, includes datasets, working papers, reports, and so on.

### 4.2 Original systematic review search strategy performance

As seen in Table 5, original systematic review search strategy performance was poor in terms of precision, with the typical review having a median precision of 0.83% (IQR: 3.29), median F1 score of 0.02 (IQR: 0.063), median F2 score of 0.04 (IQR: 0.14), and median F3 score of 0.08 (0.236). Reference search strategy performance was found to be significantly higher in terms of median precision, F1 score, F2 score, and F3 score in the *CDSR* reviews compared to the *Campbell Reviews* (Supplementary Table S5).

**Table 5:** Median (IQR) precision, F1 score, F2 score, and F3 score for all search strategies employed by the systematic reviews in the dataset

Source database	Precision % (IQR)	F1 score (IQR)	F2 score (IQR)	F3 score (IQR)
CEEDER	1.15 (3.219)	0.02 (0.062)	0.05 (0.138)	0.10 (0.231)
Campbell	0.46 (0.374)^a^	0.01 (0.007)^a^	0.02 (0.018)^a^	0.04 (0.035)^a^
CDSR	3.81 (9.408)^a^	0.07 (0.168)^a^	0.16 (0.318)^a^	0.27 (0.445)^a^
All reviews	0.83 (3.269)	0.02 (0.063)	0.04 (0.14)	0.08 (0.236)

a Significant difference between CDSR and Campbell reviews (adjusted p < 0.05).

### 4.3 Performance of automated citation searching

Overall performance of automated citation searching was poor, with median recall across all sample systematic reviews at 35.79% (IQR: 33.46%), median precision at 2.57% (IQR: 3.64%), median F1 score at 0.048 (IQR: 0.047), median F2 score at 0.031 (IQR: 0.044), and median F3 score at 0.028 (IQR: 0.040).

### 4.4 Automated citation searching vs reference search strategies

**Figure 3:**
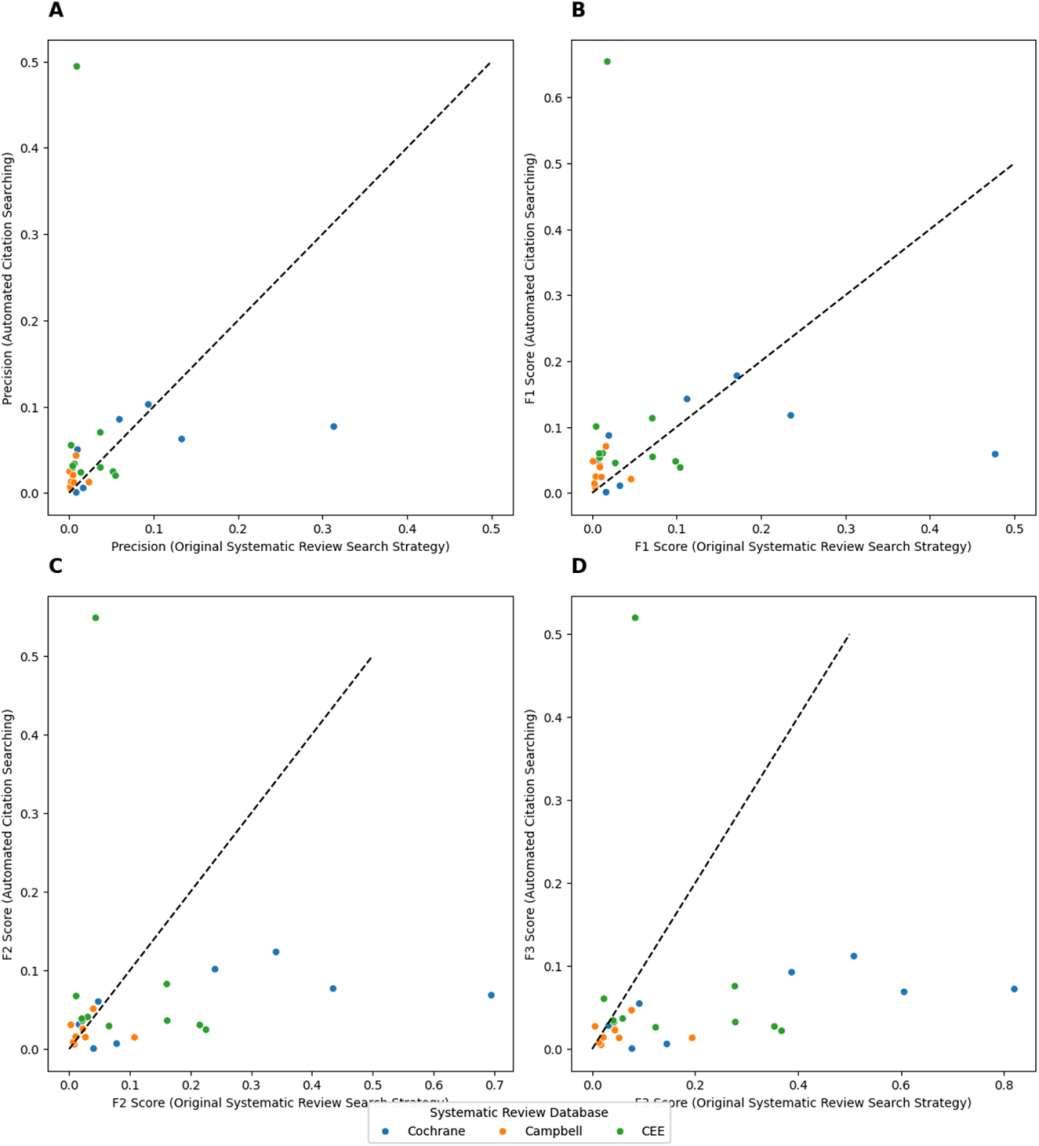
(A-D) Comparison of Automated Citation Searching Performance (Best Performing Run) vs Search Strategies employed by Sample Systematic Review, by Precision, F1 Score, F2 Score and F3 score. Observations above dotted line indicates out-peformance of automated method vs reference standard.

As pictured in Figure 3A, the automated method outperformed the reference search strategy in terms of precision in 70.4% (19/27) of cases. However, observed out-performance started to deteriorate once recall was weighted, with observed outperformance in terms of F1 score dropping to 67% (18/27) of cases (Figure 3B). This further dropped to 48.1% (13/27) of cases when recall was weighted at two times as important as precision (F2 score, Figure 3C), and finally to 11.1% (3/27) when recall was weighted as three times important as precision (F3 score, Figure 3D).

As summarized in Table 6, observed outperformance by automated citation searching vs the reference search strategy was significant in terms of precision and F1 score, with a median precision of 2.574% (IQR: 3.637) compared with 0.832% (IQR: 3.269) and a median F1 score of 0.048 (IQR: 0.047) compared to 0.016 (IQR: 0.063). However, the reference search strategy significantly outperformed in recall and F3 score (Table 6), though this assumes that the original systematic review had retrieved all possible relevant for articles for inclusion.

**Table 6:** Performance (precision, F1 score, F2 score, and F3 score) of automated citation searching vs reference systematic review search strategies

	Median	Median			
	recall %	precision %	Median F1	Median F2	Median F3
Methods	(IQR)	(IQR)	score (IQR)	score (IQR)	score (IQR)
Automated citation searching	35.789% (33.460)	2.574% (3.637)^a^	0.048 (0.047)^a^	0.031 (0.044)	0.028 (0.040)^a^
Reference systematic review search strategy (Boolean keyword search + additional supplementary strategies)	100.000% (0.000)^a^^,^ ^b^	0.832% (3.269)^a^	0.016 (0.063)^a^	0.040 (0.140)	0.077 (0.236)^a^

a Significant difference between automated citation searching and reference systematic review search strategy (p < 0.05).

b Assumes that reference systematic review had retrieved all possible relevant articles for inclusion, thus set to 100%.

## 5 Factors influencing automated citation searching performance

### 5.1 Significant factors: Study area

Among the factors examined, only study area significantly influenced automated citation searching performance, affecting precision, F1, F2, and F3 scores.

Table 7 summarizes the best performing automated citation searching runs across different APIs, categorized by systematic review subsets. While recall was highest in the *CEEDER* subset, followed by *Campbell* and *CDSR*, the observed variation across these subsets was not significant. In terms of precision, F1, F2, and F3 scores, performance was highest in the CDSR subset, followed by *CEEDER*, and then *Campbell*. The differences were significant between the *CEEDER* and *Campbell* subsets, as detailed in Table 7.

**Table 7:** Median (IQR) recall, precision, F1 score, F2 score, and F3 score of the best performing automated citation searching runs, by systematic review subsets

Systematic					
review	Median (IQR)	Median (IQR)	Median (IQR)	Median (IQR)	Median (IQR)
subset	recall (%)	precision (%)	F1 score	F2 score	F3 score
All reviews^a^	35.79 (33.50)	2.57 (3.60)	0.048 (0.047)	0.031 (0.044)	0.028 (0.040)
CEEDER	52.47 (33.80)	3.05 (2.40)^b^	0.057 (0.041)^b^	0.037 (0.029)^b^	0.034 (0.026)^b^
Campbell	35.48 (12.50)	1.28 (1.00)^b^	0.025 (0.020)^b^	0.016 (0.012)^b^	0.014 (0.011)^b^
CDSR	30.74 (27.10)	5.63 (5.80)	0.073 (0.086)	0.064 (0.058)	0.062 (0.055)

a Aggregate of all systematic reviews.

b Significant difference between CEEDER and Campbell subsets (adjusted p < 0.05).

### 5.2 Nonsignificant factors

Other examined factors did not show significant effects. However, there were some interesting trends as summarized in Tables S3-S4 in the Supplementary Material.

First, automated citation searching tended to perform better on sample systematic reviews that only included the peer-reviewed literature compared to systematic reviews that included both the peer reviewed literature and the gray literature, with higher median recall, precision, and F scores. In terms of seed article types, framework and consensus articles yielded the top two highest median recall scores at 29.55% (IQR: 20.50) and 23.33% (IQR: 10.00), respectively. This was followed by “other” articles, methodology articles, research articles, evidence synthesis articles, and lastly commentary articles. However, once precision was weighted through the F scores, commentary articles merged as the leading type of seed article, followed by evidence synthesis articles, framework articles, “other” articles, methodology articles, and lastly consensus articles. There were marginal differences in performances between the two APIs tested, with automated citation searching runs through the Semantic Scholar API exhibiting a higher median recall and F1 score compared to the API. However, runs from the OpenAlex API tended to exhibit higher precision, F2 scores and F3 scores, respectively.

Additionally, Intracluster semantic similarity tended to show moderate positive correlation with recall, and weak positive correlations with all other performance measures. On the other hand, the number of seed articles used in a particular automated searching run was found to have limited correlation with recall and was negatively correlated with all other performance measures. Lastly, the number of included articles extracted per sample systematic review also showed weak negative correlations with precision, and limited correlation with all other performance metrics.

### 5.3 Baseline retrievability of included articles

As illustrated in Table 8, the median percentage of included articles with valid IDs in the typical systematic review was 86.4% (IQR: 12.05%). Systematic reviews in the *Campbell* subset had the highest median percentage of valid IDs, followed by *CDSR* and *CEEDER*, yet the differences were not statistically significant.

**Table 8:** Median % (IQR) of included articles with Valid IDs extracted from systematic reviews in dataset, and baseline retrievability rate of included articles across both APIs: (OpenAlex, Semantic Scholar)

	Median % (IQR) of	Median (IQR) baseline	Median (IQR) baseline
	included articles with	retrievability rate %	retrievability rate %
Source database	valid IDs^a^	(OpenAlex)	(Semantic Scholar)
All reviews	86.4 (12.05)	85.7 (13.2)	85.7 (16.4)
CEEDER	84.4 (15.52)	81.6 (15.52)	83.35 (17.65)
Campbell	89.1 (6.3)	86.4 (4.6)	87.0 (4.6)
CDSR	85.9 (15.2)	85.9 (15.2)	84.0 (18.23)

a Valid IDs refer to PMIDs, DOI, or MAG IDs.

However, the baseline retrievability rate of included articles across each API (OpenAlex and Semantic Scholar) were lower than the percentage of included articles that had valid IDs, indicating potential deficits in database coverage. Differences in retrieval rates across both APIs were nonsignificant.

### 5.4 Automated citation searching performance across benchmarks

**Figure 4:**
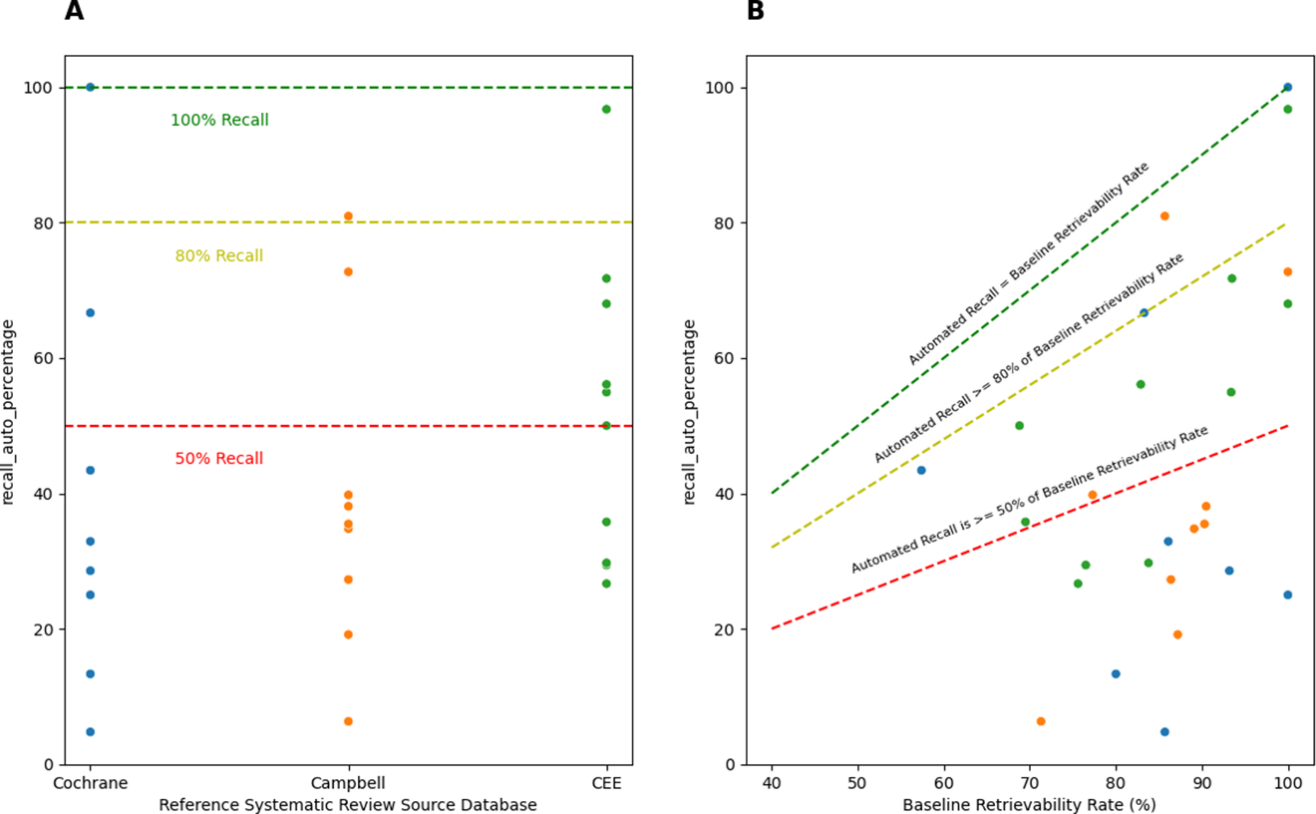
Recall of automated citation searching for each systematic review against various level of recall (A), and against the baseline retrievability rate of included articles of each systematic review (B).

Figure 4A compares the recall of the best performing automated citation searching run, irrespective of API, against three recall thresholds: 50%, 80%, and 100%. As shown, 100% recall was achieved for only 1 case, the 80% threshold was exceeded in 11.1% (3/27) of cases, and the 50% recall threshold was exceeded in 37% (10/27) of cases.

Similarly, Figure 4B compares recall against the baseline retrievability rate of included articles. Recall matched the baseline rate in only one case and exceeded the 80% threshold in 14.8% (4/27) of cases and the 50% threshold in 40.7% (11/27) of cases, suggesting potential for improvement in the automated technique.

## 6 Discussion

To our knowledge, this is the first study of its kind that investigates performance differences in automated citation searching across different study areas and further investigates potential factors that influence performance. Additionally, prior related simulation studies utilized different variants of automated citation searching, ranging from co-citation variants,^15^ to citation clusters,^16^ with most focus on the biomedical literature.^16^^,^ ^17^ Additionally, prior related work has utilized different databases, ranging from the Web of Science (WOS),^15^ to Lens.org,^9^ to the Dimensions database,^16^ and lastly PubMed.^10^ Our work here investigates the use of both the OpenAlex and Semantic Scholar APIs. However, none of the simulation studies^15^^–^ ^17^ had investigated the variant of automated citation searching as investigated here, where only the citation network within 1 “hop” of a seed article is retrieved.

### 6.1 Principal findings

Our results indicate that automated citation searching offers improved precision but struggles with recall compared to traditional methods. Thus, while it is efficient at retrieving relevant articles, it may miss a significant number of articles that would be found by conventional methods. As such, automated citation searching should be best used as a supplementary search strategy in traditional systematic review production, or as an initial scoping search tool for resource-constrained settings. As a standalone method, there is a risk of missing potentially relevant literature, and its suitability decreases as the need for capturing all of the relevant literature increases. This is evidenced by both poor recall, and F3 score (weighted average between recall and precision, where recall is 3x as important as precision). However, observed outperformance in F1 score (weighted average between recall and precision, where recall is 1x as important as precision) indicates that its integration as a supplementary method would not adversely affect the downstream workload when it comes to the screening process.

Performance of automated citation searching was study area dependent, notably with performance in terms of precision, F1 score, F2 score, and F3 score being significantly higher within the environmental management literature (as represented by systematic reviews from *CEEDER*) relative to the social policy literature, as represented by systematic reviews from *Campbell* reviews.

### 6.2 Study area and influence on performance

We hypothesize that observed performance differences across study areas may stem from i) varying levels of consensus on concepts, terms, and definitions, ii) differences in research question broadness, or iii) a combination of both. Authors in areas with high consensus are more likely to cite the same articles, resulting in citation networks that are more likely to yield relevant articles. This could result in enhanced automated citation searching performance. Despite a limited sample size, the high recall of Consensus and Framework articles as seed article types supports this observation (Table 8).

We further note that the *CDSR* (Cochrane) subset exhibited the lowest median recall and highest F3 score. This could possibly be due to the diverse range of research questions that were the subject of the Cochrane reviews in the dataset, ranging from health equity assessments^18^ to clinical interventions.^19^ Furthermore, the nonretrievability of clinical trials and conference abstracts by both OpenAlex and Semantic Scholar APIs may have contributed to poorer performance in the Cochrane subset.

### 6.3 Limitations of automated citation searching

Current methods of automated citation searching relies heavily on unique identifiers such as DOIs,^9^ PMIDs,^9^^,^ ^10^ and MAG IDs^9^ to identify, disambiguate, and retrieve articles. This method is further constrained by the coverage of APIs that provide access to these IDs and citation links necessary for building citation networks. We noted through the course of our research that the absence of valid DOIs and other IDs primarily came from grey literature, clinical trial reports and conference abstracts. This puts a theoretical limit on the performance that is achievable via automated citation searching and other methods that rely on such unique identifiers. Nonetheless, as indicated by Figure 5B, the gap between the current performance of automated citation searching and what can be theoretically achieved suggests that more sophisticated methods, such as co-citations,^15^^,^ ^16^ additional “hops” through the citation network beyond just 1 “hop” as tested in this study,^20^ and vector-based retrieval strategies,^21^ can yield further improvements in performance. Additionally, whilst this was not investigated in the current study, the number of backwards and forwards citations of a seed article may also influence the final performance of the technique, and future work should investigate where this should be a factor in seed article selection.

### 6.4 Recommendations for current use

Despite its limitations as a standalone method, automated citation searching still offers distinct advantages in terms of speed, replicability, and convenience. However, there are a dearth of publicly available and accessible tools to allow for adoption. To this end, a publicly available web-app^22^ leveraging the databases tested in this study (OpenAlex and Semantic Scholar) is available for use and further testing: https://darrenkjr-automatedcitationsearch.streamlit.app/. Our findings suggest that automated citation searching may be best used as a supplementary strategy in study areas with high consensus on research direction, diagnostic criteria, or definitions. Selection of seed articles should reflect such consensus, using articles like core outcome sets and diagnostic criteria as “signposts.” Automated citation searching may also have potential in resource constrained contexts where recall is as important as precision, such as in rapid reviews or surveillance searches in the context of living guideline updates.^7^

### 6.5 System-level approaches toward improving automated citation searching performance

Our work suggests that the performance of automated citation searching is currently limited by technical aspects related to API coverage and socioecological aspects related to the citation activity of authors. From an API coverage perspective, better support for grey literature and clinical trial identification may improve performance, alongside improvements to data processing. Current methods rely on the parsing of full text PDFs to extract citation links, which is non-trivial. An alternative would be the development of an alternative format for journal article publishing built for interoperability in terms of data sharing and machine readability. For example, such has been done with digital health via the Fast Healthcare Interoperability Resources (FHIR) standard^23^ and has recently proposed by Haddaway et al.^24^ for systematic reviews and other evidence syntheses. From a socioecological perspective, increased adoption of consensus building activities within fields such as core outcome sets and evidence-based guidelines may also yield further improvements in downstream automated citation searching performance, beyond improvements in tackling research waste and research transparency.^25^

### 6.6 Future directions

Our work evaluates a simple form of automated citation searching in evidence synthesis and conducts an exploratory investigation into the situations and contexts where such methods may be best deployed. A publicly available webapp has been developed through this to allow for further testing in different contexts.^22^ Despite poor performance in recall, preliminary results indicate that there is further scope for improvement on the technique particularly with other variants having shown promising results in the biomedical literature specifically.^5^^,^ ^15^^,^ ^16^ More work with a larger sample size investigating potential performance factors such as the citation network size of seed articles, and sensitivity analyses investigating the effect of using included articles as seed articles is also warranted to further optimize the technique and produce empirically derived guidance. However, for automated evidence synthesis to truly gain mainstream adoption, more efforts are needed to integrate what is currently a disparate tool chain with high technical hurdles for adoption; into a more user-friendly interface, crucially starting from the beginning of the evidence synthesis process, specifically the scoping and search strategy development phase. Future directions should be focused on current tool integration, combining automated evidence retrieval with automated title and abstract screening, and evaluating such tools across a diverse set of contexts and study areas. Additionally, as the support of more databases requires technical expertise, open-source efforts to pool resources should be encouraged to allow for greater user choice, and lower the technical gap to access such tools.

### 6.7 Limitations of the study

Our work here assumed that all the sample systematic reviews had retrieved all possible relevant articles that were eligible for inclusion when the original search was conducted. In practice, some eligible articles may have been missed by the original search, and the performance of the original search strategies might be overinflated. It is also possible that automated citation searching may have retrieved articles which may have been overlooked in the original systematic review due to the differences in coverage between the APIs and the databases employed by the systematic reviews. As such, recall for automated citation searching may have been underestimated. Further, both OpenAlex and Semantics Scholar have bespoke ID systems beyond the ID types that were used in this study. As such, included articles that may have been retrievable in either API may not have been uncovered, thus underestimating recall. Lastly, our seed article selection strategy leveraged articles from the Background and Methods sections of each sample systematic review, assuming the worst-case scenario where systematic reviews have no prior knowledge of included articles that could be relevant to the review question, with potentially no included articles that could be relevant. In reality, leveraging included articles could yield better results. As such, our results could be potentially underestimating its efficacy.

## 7 Conclusion

Automated citation searching is currently best used as a supplementary search strategy during evidence synthesis and systematic review production due to poor performance in terms of recall (captured less relevant articles compared to standard practice). However, it outperforms standard methods in terms of precision (proportion of relevant articles identified from all articles identified was better than standard practice). As a result, it may have other niche applications in initial scoping searches or rapid reviews. However, its suitability decreases as the need for higher recall increases, as evidenced by its poor performance in terms of F3 score (where recall is weighted 3x as important as precision). Nonetheless, it can be potentially integrated as a supplementary method without overly burdening the screening process as evidenced by its higher F1 score (where recall is as important as precision) relative to conventional methods. Lastly, the performance of automated citation searching is dependent on study area, potentially due to differing levels of consensus on aspects such as diagnostic criteria, research directions, and term definitions. As such, seed article choice in automated citation searching should take this aspect into account.

## Supporting information

Rajit et al. supplementary material Rajit et al. supplementary material

## Data Availability

The data that support the findings of this study are openly available in a publicly available Github repository: https://github.com/darrenkjr/automated_citation_search_study

## Supplementary material

To view supplementary material for this article, please visit http://doi.org/10.1017/rsm.2024.15.
