# Supplementary material for: Automated citation searching in systematic review production: A simulation study
Source: Res Synth Methods. 2025 Mar 7;16(1):211–27. doi: 10.1017/rsm.2024.15 (PMC12621532; doi:10.1017/rsm.2024.15)
Supplement: Rajit et al. supplementary material [file S1759287924000152sup001.docx]

# Extensions form Initial Protocol

### Expanded Systematic Review Baseline Characteristics Extraction

Firstly, whilst all reviews employed a Boolean-based search strategy as the main evidence retrieval method, there was diversity in the supplementary search strategies (Table S1). Inclusion criterion varied between systematic reviews as well, specifically regarding the inclusion of peer-reviewed literature vs grey literature. As such, we expanded baseline characteristic extraction to encompass all search strategies and study type.

### Assessing Baseline Retrievability of Included Articles

We also assessed the maximum percentage of included papers that were retrievable by automated citation searching, as some lacked valid IDs such as DOIs, PMIDs, or MAG IDs. This limitation imposed a ceiling on automated retrievability of articles. We reported two key metrics: the percentage of papers with retrievable IDs and the baseline retrievability of included articles for each API (OpenAlex / Semantic Scholar). Baseline retrievability is defined as the percentage of included articles successfully retrieved from the API.

### Refined Seed Article Selection

To reduce bias, we refined the criteria for selecting seed articles. We excluded articles from the candidate pool that were both eligible for inclusion and had been referenced in the background and methods sections of the sample systematic reviews. This approach simulates the scenario where review teams are unaware of included articles *a priori.* Due to API limitations and computational constraints, we excluded seed article candidates with over 10,000 citations. These highly-cited articles, typically statistical methods and evidence synthesis methods, were unlikely to affect results.

### Revised Seed Article Study Type Classification

The original classification typology for seed articleswere also modified.. The categories “Secondary Articles” and “Primary Articles” were removed and replaced by the categories of “Research Article” and “Evidence Synthesis”. “Research article” are individual studies that either use primary or secondary data, whereas “Evidence Syntheses” are articles that are meant to summarize the evidence for a particular subject area, either through narrative means as Narrative Reviews, or through structured and systematic methods, such as Systematic Reviews, Scoping Reviews, Evidence Gap Maps and Meta-Analyses. This was required as the prior typology grouped reviews and research articles employing secondary data together.

### Best Automated Citation Searching Run Selection

The original protocol lacked provisions for selecting the best automated citation searching run in the case of recall score ties. F3 score was added as a tiebreaker in this instance to balance precision and recall. Given F3 score emphasizes recall over precision by a factor of 3, it serves as a proxy for the real world need to balance the need to capture all possible relevant evidence (maximize recall), and resource constraints when proceeding to the screening stage (maintain reasonable precision).

### Automated Citation Searching Performance vs Benchmarks

To contextualize automated citation searching in real-world scenarios, we also compared its performance against three recall levels (50%, 80%, 100%) and the baseline retrievability of included studies. We chose varying recall levels to represent different evidence synthesis scenarios, from pragmatic rapid reviews tolerating lower recall due to resource constraints to full systematic reviews prioritizing maximal recall. Additionally, since the baseline retrievability of included articles sets a theoretical maximum for recall achievable by automated methods, our comparisons aim to determine how close our approach comes to this limit, highlighting potential areas for improvement with more advanced automated techniques

# Supplementary Figures


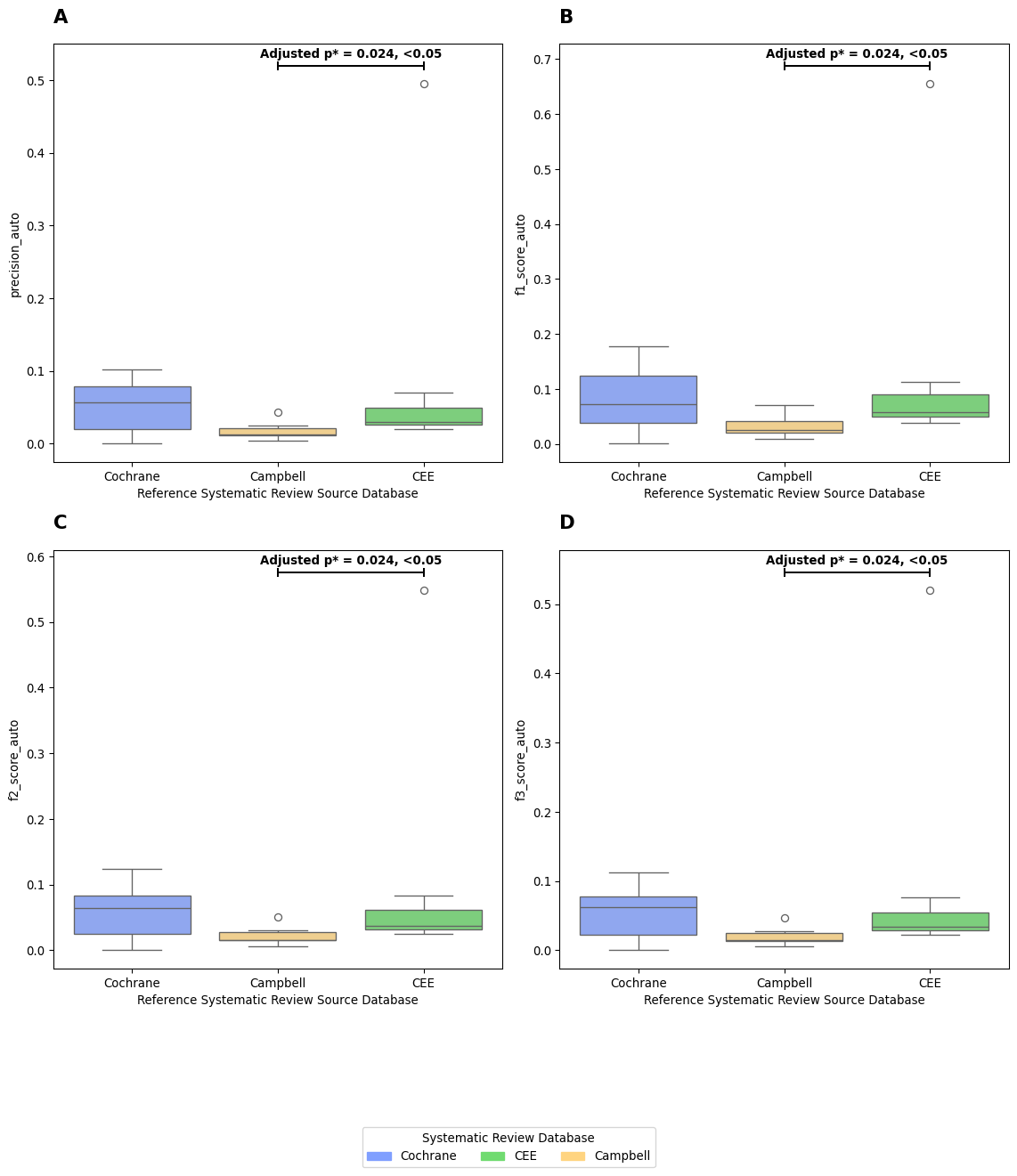


Figure S1: (A-D) Comparison of Automated Citation Searching Performance (Best Performing Run) within different Systematic Review Subsets, by Precision, F1 Score, F2 Score and F3 score.

| 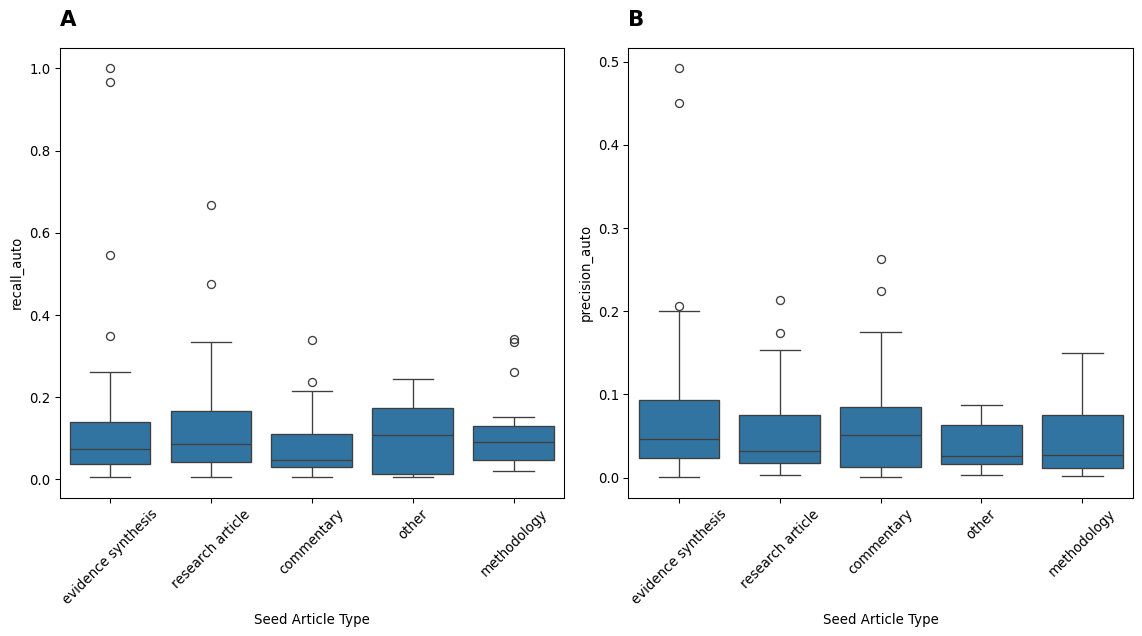  Figure S2: Box plot of various seed article types vs recall (A) and precision (B). Only seed article types with n>5 are plotted. |
| --- |

| 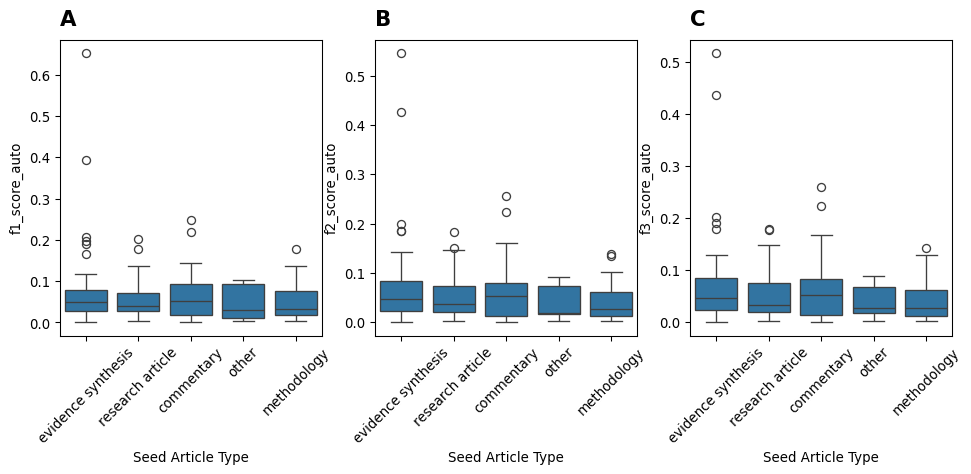  Figure S3: Box plot of various seed article types vs F-1 Score (A) F2 Score (B) and (C) F3 Score. Only seed article types with n>5 are plotted. |
| --- |

| 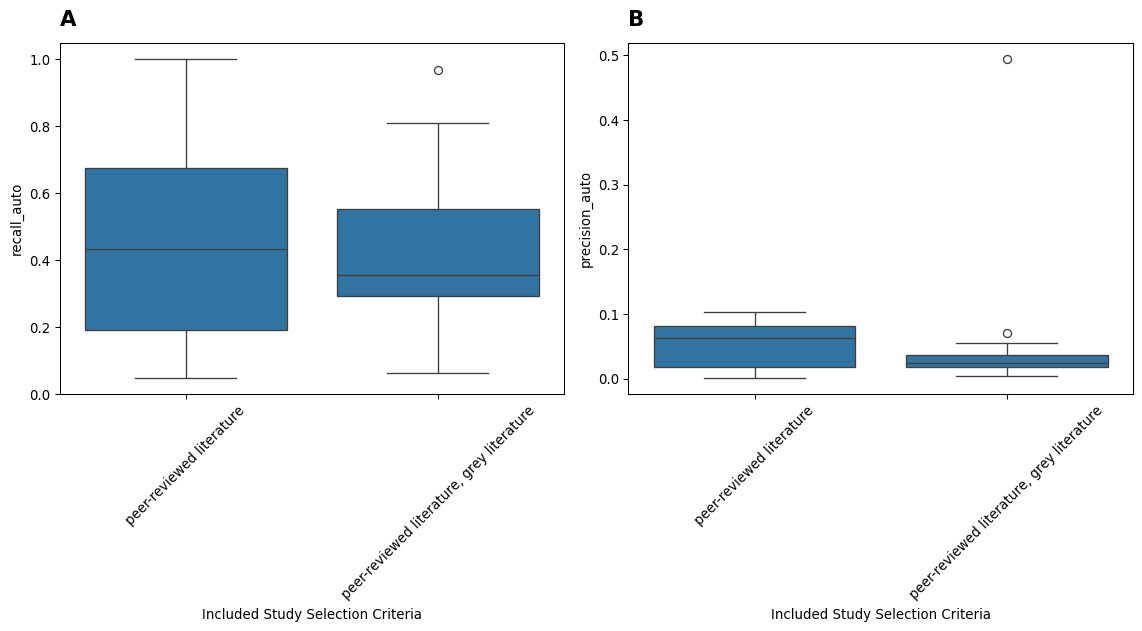  Figure S4: Boxplot of (A) Recall and (B) Precision, by study type inclusion criteria |
| --- |

| 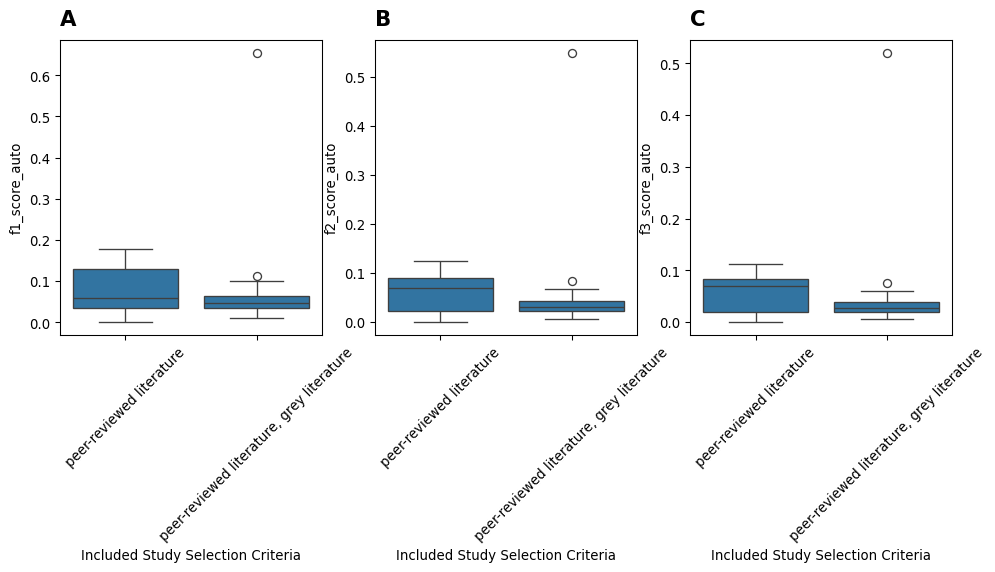  Figure S5: Boxplot of F1 score, F2 score and F3 score by study type inclusion criteria |
| --- |

| 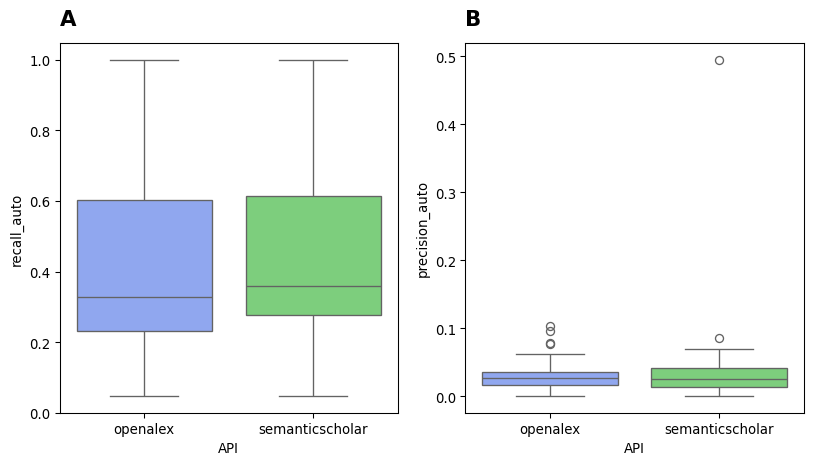  Figure S6: Boxplot of (A) Recall and (B) Precision of the best performing automated citation searching runs for each API |
| --- |

| 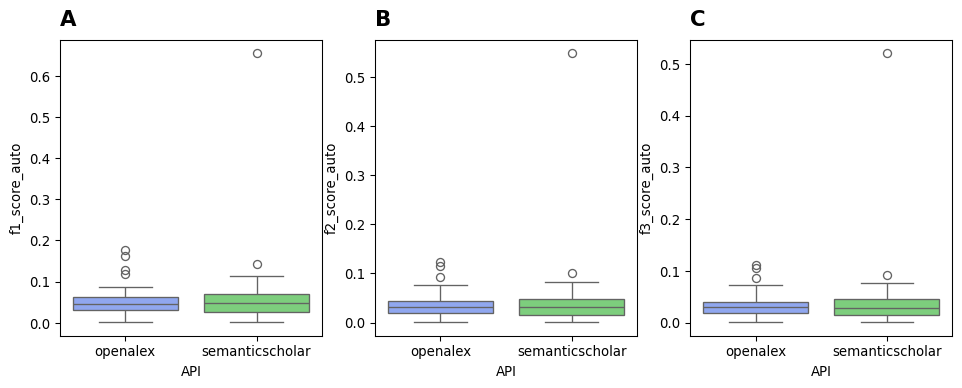  Figure S7: Boxplot of (A) F1 Score, (B) F2 Score, and (C) F3 Score of the best performing automated citation searching runs for each API |
| --- |

# Supplementary Tables

**Table S1 : Sample Systematic Review Characteristics (Title, Source Database, Publication Year, Search Strategy, Study Type Inclusion Criteria)**

| # | Title | Source Database | Publication Year | Search Strategy | Study Type Inclusion Criteria |
| --- | --- | --- | --- | --- | --- |
| 1 | Mechanisms of Impact of Blue Spaces on Human Health: A Systematic Literature Review and Meta-Analysis | CEEDER | 2021 | boolean keyword + backwards citation search | peer-reviewed literature |
| 2 | A systematic review of the socio-economic impacts of large-scale tree plantations, worldwide | CEEDER | 2018 | boolean keyword + backwards citation search + handsearch | peer-reviewed literature, grey literature |
| 3 | Are small protected habitat patches within boreal production forests effective in conserving species richness, abundance and community composition? A systematic review | CEEDER | 2021 | boolean keyword + full citation search + handsearch + expert consultation | peer-reviewed literature, grey literature |
| 4 | What is the effect of phasing out long-chain per- and polyfluoroalkyl substances on the concentrations of perfluoroalkyl acids and their precursors in the environment? A systematic review | CEEDER | 2018 | boolean keyword + handsearch + expert consultation | peer-reviewed literature, grey literature |
| 5 | How does roadside vegetation management affect the diversity of vascular plants and invertebrates? A systematic review | CEEDER | 2018 | boolean keyword + prior evidence map | peer-reviewed literature, grey literature |
| 6 | The effectiveness of non-native fish removal techniques in freshwater ecosystems: a systematic review | CEEDER | 2019 | boolean keyword + backwards citation search + handsearch + expert consultation + crowdsourcing | peer-reviewed literature, grey literature |
| 7 | Impacts of dead wood manipulation on the biodiversity of temperate and boreal forests. A systematic review | CEEDER | 2019 | boolean keyword + handsearch + prior evidence map | peer-reviewed literature, grey literature |
| 8 | The Different Dimensions of Livelihood Impacts of Payments for Environmental Services (PES) Schemes: A Systematic Review | CEEDER | 2018 | boolean keyword | peer-reviewed literature, grey literature |
| 9 | The effectiveness of spawning habitat creation or enhancement for substrate-spawning temperate fish: a systematic review | CEEDER | 2019 | boolean keyword + backwards citation search + handsearch + crowdsourcing | peer-reviewed literature, grey literature |
| 10 | What are the effects of even-aged and uneven-aged forest management on boreal forest biodiversity in Fennoscandia and European Russia? A systematic review | CEEDER | 2021 | boolean keyword + full citation search + handsearch | peer-reviewed literature, grey literature |
| 11 | Strengthening women’s empowerment and gender equality in fragile contexts towards peaceful and inclusive societies: A systematic review and meta‐analysis | Campbell Reviews | 2022 | boolean keyword + full citation search + handsearch | peer-reviewed literature, grey literature |
| 12 | Red light camera interventions for reducing traffic violations and traffic crashes: A systematic review | Campbell Reviews | 2020 | boolean keyword + backward citation search + handsearch | peer-reviewed literature, grey literature |
| 13 | Aquaculture for improving productivity, income, nutrition and women’s empowerment in low‐ and middle‐income countries: A systematic review and meta‐analysis | Campbell Reviews | 2021 | boolean keyword + full citation search + handsearch | peer-reviewed literature, grey literature |
| 14 | Policies and interventions to remove gender‐related barriers to girls’ school participation and learning in low‐ and middle‐income countries: A systematic review of the evidence | Campbell Reviews | 2022 | boolean keyword + backwards citation search + handsearch | peer-reviewed literature, grey literature |
| 15 | Citizen engagement in public services in low‐ and middle‐income countries: A mixed‐methods systematic review of participation, inclusion, transparency and accountability (PITA) initiatives | Campbell Reviews | 2019 | boolean keyword + backwards citation search + handsearch | peer-reviewed literature, grey literature |
| 16 | The impacts of agroforestry interventions on agricultural productivity, ecosystem services, and human well‐being in low‐ and middle‐income countries: A systematic review | Campbell Reviews | 2021 | boolean keyword + full citation search + handsearch + prior evidence map | peer-reviewed literature, grey literature |
| 17 | Multiagency programs with police as a partner for reducing radicalisation to violence | Campbell Reviews | 2021 | boolean keyword + full citation search + expert consultation | peer-reviewed literature, grey literature |
| 18 | Adult/child ratio and group size in early childhood education or care to promote the development of children aged 0–5 years: A systematic review | Campbell Reviews | 2022 | boolean keyword + full citation search + handsearch + expert consultation | peer-reviewed literature, grey literature |
| 19 | Interventions for improving executive functions in children with foetal alcohol spectrum disorder (FASD): A systematic review | Campbell Reviews | 2022 | boolean keyword + full citation search + handsearch + expert consultation | peer-reviewed literature, grey literature |
| 20 | Selective serotonin reuptake inhibitors (SSRIs) for stroke recovery | CDSR | 2021 | boolean keyword + backwards citation search + expert consultation | peer-reviewed literature |
| 21 | Vena caval filters for the prevention of pulmonary embolism | CDSR | 2020 | boolean keyword + forward citation + previous version of review | peer-reviewed literature |
| 22 | Atovaquone-proguanil for treating uncomplicated Plasmodium falciparum malaria | CDSR | 2021 | boolean keyword + backwards citation search + previous version of review | peer-reviewed literature |
| 23 | Pharmaceutical policies: effects of regulating drug insurance schemes | CDSR | 2022 | boolean keyword + backwards citation search + expert consultation | peer-reviewed literature, grey literature |
| 24 | Ab interno supraciliary microstent surgery for open-angle glaucoma | CDSR | 2021 | boolean keyword + backwards citation search + handsearch | peer-reviewed literature |
| 25 | Probiotics for the prevention of Hirschsprung-associated enterocolitis | CDSR | 2022 | boolean keyword + backwards citation search + expert consultation | peer-reviewed literature |
| 26 | Pentoxifylline for the treatment of endometriosis-associated pain and infertility | CDSR | 2021 | boolean keyword + backwards citation search + expert consultation | peer-reviewed literature |
| 27 | How effects on health equity are assessed in systematic reviews of interventions | CDSR | 2022 | boolean keyword + backwards citation search + expert consultation + previous version of review | peer-reviewed literature, grey literature |

**Table S2: Percentage of Included Articles with Retrieval IDs, and corresponding theoretical maximum achievable recall for each API (OpenAlex and Semantic Scholar), for each Systematic Review**

|  | Source Database | Number of Included Articles | Percentage of Included Articles with Retrievable IDs | Maximum Theoretical Recall (OpenAlex) | Maximum Theoretical Recall (Semantic Scholar) |
| --- | --- | --- | --- | --- | --- |
| 0 | CEEDER | 50 | 100.0 % (n=50) | 100.0 % (n=50) | 96.0 % (n=48) |
| 1 | CEEDER | 90 | 77.8 % (n=70) | 77.8 % (n=70) | 75.6 % (n=68) |
| 2 | CEEDER | 41 | 82.9 % (n=34) | 80.5 % (n=33) | 82.9 % (n=34) |
| 3 | CEEDER | 92 | 100.0 % (n=92) | 100.0 % (n=92) | 100.0 % (n=92) |
| 4 | CEEDER | 51 | 78.4 % (n=40) | 78.4 % (n=40) | 76.5 % (n=39) |
| 5 | CEEDER | 95 | 69.5 % (n=66) | 66.3 % (n=63) | 69.5 % (n=66) |
| 6 | CEEDER | 91 | 93.4 % (n=85) | 93.4 % (n=85) | 93.4 % (n=85) |
| 7 | CEEDER | 46 | 93.5 % (n=43) | 93.5 % (n=43) | 93.5 % (n=43) |
| 8 | CEEDER | 64 | 70.3 % (n=45) | 67.2 % (n=43) | 68.8 % (n=44) |
| 9 | CEEDER | 185 | 85.9 % (n=159) | 82.7 % (n=153) | 83.8 % (n=155) |
| 10 | Campbell | 21 | 90.5 % (n=19) | 85.7 % (n=18) | 90.5 % (n=19) |
| 11 | Campbell | 110 | 92.7 % (n=102) | 90.9 % (n=100) | 86.4 % (n=95) |
| 12 | Campbell | 47 | 87.2 % (n=41) | 85.1 % (n=40) | 87.2 % (n=41) |
| 13 | Campbell | 88 | 86.4 % (n=76) | 86.4 % (n=76) | 77.3 % (n=68) |
| 14 | Campbell | 46 | 89.1 % (n=41) | 89.1 % (n=41) | 87.0 % (n=40) |
| 15 | Campbell | 11 | 100.0 % (n=11) | 100.0 % (n=11) | 100.0 % (n=11) |
| 16 | Campbell | 174 | 77.6 % (n=135) | 71.3 % (n=124) | 76.4 % (n=133) |
| 17 | Campbell | 31 | 93.5 % (n=29) | 90.3 % (n=28) | 90.3 % (n=28) |
| 18 | Campbell | 21 | 85.7 % (n=18) | 85.7 % (n=18) | 85.7 % (n=18) |
| 19 | Cochrane | 129 | 59.7 % (n=77) | 59.7 % (n=77) | 57.4 % (n=74) |
| 20 | Cochrane | 15 | 80.0 % (n=12) | 80.0 % (n=12) | 80.0 % (n=12) |
| 21 | Cochrane | 21 | 85.7 % (n=18) | 85.7 % (n=18) | 85.7 % (n=18) |
| 22 | Cochrane | 79 | 86.1 % (n=68) | 86.1 % (n=68) | 82.3 % (n=65) |
| 23 | Cochrane | 4 | 100.0 % (n=4) | 100.0 % (n=4) | 100.0 % (n=4) |
| 24 | Cochrane | 2 | 100.0 % (n=2) | 100.0 % (n=2) | 100.0 % (n=2) |
| 25 | Cochrane | 6 | 83.3 % (n=5) | 83.3 % (n=5) | 66.7 % (n=4) |
| 26 | Cochrane | 161 | 96.9 % (n=156) | 96.9 % (n=156) | 93.2 % (n=150) |

**Table S3: Median (IQR) Recall, Precision, F1 score, F2 score, F3 score by study type inclusion criteria, best performing seed article type, and API used**

| **Factors** | | **Median (IQR) Recall (%)** | **Median (IQR) Precision (%)** | **Median (IQR) F1 Score** | **Median (IQR) F2 Score** | **Median (IQR) F3 Score** |  |
| --- | --- | --- | --- | --- | --- | --- | --- |
| **Study Type Inclusion Criteria (n)** | | | | | | | |
|  | Peer-reviewed literature (7) | 43.41 (48.20) | 6.25 (6.30) | 0.060 (0.095) | 0.068 (0.066) | 0.069 (0.062) |  |
|  | Peer-reviewed literature & grey literature (20) | 35.64 (26.00) | 2.48 (1.80) | 0.047 (0.028) | 0.031 (0.021) | 0.027 (0.019) |  |
| **Seed Article Type (n)** | | | | | | | |
|  | Framework (2) | 29.55 (20.50) | 2.55 (0.10) | 0.044 (0.003) | 0.031 (0.000) | 0.028 (0.001) |  |
|  | Consensus article (2) | 23.33 (10.00) | 1.80 (1.20) | 0.033 (0.022) | 0.022 (0.015) | 0.020 (0.014) |  |
|  | Other§ (9) | 10.87 (16.10) | 2.55 (4.70) | 0.030 (0.083) | 0.020 (0.055) | 0.028 (0.050) |  |
|  | Methodology (17) | 9.09 (8.10) | 2.68 (6.40) | 0.033 (0.057) | 0.026 (0.048) | 0.027 (0.049) |  |
|  | Research article (32) | 8.61 (12.40) | 3.24 (5.70) | 0.040 (0.044) | 0.037 (0.052) | 0.034 (0.056) |  |
|  | Evidence synthesis (37) | 7.32 (10.40) | 4.69 (7.10) | 0.049 (0.051) | 0.047 (0.060) | 0.047 (0.061) |  |
|  | Commentary (24) | 4.72 (8.10) | 5.07 (7.20) | 0.053 (0.076) | 0.054 (0.066) | 0.052 (0.070) |  |
| **API** | | | | | | | |
|  | OpenAlex | 32.91 (37.10) | 2.75 (2.00) | 0.045 (0.033) | 0.032 (0.025) | 0.030 (0.022) |  |
|  | Semantic Scholar | 35.79 (33.60) | 2.57 (2.80) | 0.048 (0.044) | 0.031 (0.033) | 0.028 (0.030) |  |

§*Grey literature, includes datasets, working papers, reports, etc.*

**Table S4: Kruskal Wallis test results for study area vs performance metrics (original systematic review search strategy)**

| **Metric** | **Kruskal Statistic** | **Kruskal p-value (Raw)** | **Kruskal p-value (Adjusted)** |
| --- | --- | --- | --- |
| precision_percentage | 9.14 | 0.010* | 0.010 * |
| f1_score | 9.14 | 0.010* | 0.010 * |
| f2_score | 9.14 | 0.010* | 0.010 * |
| f3_score | 9.14 | 0.010* | 0.010 * |

**Table S5: Mann Whitney U test results for study area vs performance metrics (original systematic review search strategy)**

| **Metric** | **Comparison** | **U Statistic** | **Raw p-value** | **Adjusted p-value** |
| --- | --- | --- | --- | --- |
| precision_percentage | CEE vs Campbell | 71.0 | 0.037* | 0.112 |
| precision_percentage | CEE vs Cochrane | 24.0 | 0.173 | 0.518 |
| precision_percentage | Campbell vs Cochrane | 8.0 | 0.006* | 0.017* |
| f1_score | CEE vs Campbell | 71.0 | 0.037* | 0.112 |
| f1_score | CEE vs Cochrane | 24.0 | 0.173 | 0.518 |
| f1_score | Campbell vs Cochrane | 8.0 | 0.006* | 0.017* |
| f2_score | CEE vs Campbell | 71.0 | 0.037* | 0.112 |
| f2_score | CEE vs Cochrane | 24.0 | 0.173 | 0.518 |
| f2_score | Campbell vs Cochrane | 8.0 | 0.006* | 0.017* |
| f3_score | CEE vs Campbell | 71.0 | 0.037* | 0.112 |
| f3_score | CEE vs Cochrane | 24.0 | 0.173 | 0.518 |
| f3_score | Campbell vs Cochrane | 8.0 | 0.006* | 0.017* |

**Table S6: Spearman rank correlation coefficients) for intracluster semantic similarity, number of included articles, number of seed articles, against performance metrics (Recall, Precision, F1 Score, F2 score and F3 score)**

| **Factors** | **Recall** | **Precision** | **F1 Score** | **F2 Score** | **F3 Score** |
| --- | --- | --- | --- | --- | --- |
| Intracluster Semantic Similarity | 0.403 | 0.158 | 0.174 | 0.165 | 0.152 |
| Number of Included Articles | -0.231 | 0.106 | 0.096 | 0.097 | 0.111 |
| Number of Seed Articles | -0.071 | -0.296 | -0.276 | -0.295 | -0.292 |

**Table S7: Comparisons between automated citation searching vs sample systematic review search strategy performance**

| Metric | Automated Citation Searching Performance | Sample Systematic Review Performance | P-value |
| --- | --- | --- | --- |
| Recall | 35.789% (33.460) | 100.000% (0.000)^+^ | 0.000* |
| Precision | 2.574% (3.637) | 0.832% (3.269) | 0.020* |
| F1 Score | 0.048 (0.047) | 0.016 (0.063) | 0.031* |
| F2 Score | 0.031 (0.044) | 0.040 (0.140) | 0.378 |
| F3 Score | 0.028 (0.040) | 0.077 (0.236) | 0.002* |
| **p<0.05 (significant) ^+^Assumes that original systematic review had retrieved all possible relevant articles for inclusion, thus set a prior to 100%* | | |  |

**Table S8: Kruskal Wallis test results for Study Area vs Performance (Recall, Precision, F1 Score, F2 Score, F3 Score)**

| Metric | Kruskal Statistic | Kruskal p-value (Raw) | Kruskal p-value (Adjusted) |
| --- | --- | --- | --- |
| recall_auto | 1.99 | 0.370 | 0.370 |
| precision_auto | 6.88 | 0.032* | 0.032 * |
| f1_score_auto | 6.43 | 0.040* | 0.040 * |
| f2_score_auto | 6.88 | 0.032* | 0.032 * |
| f3_score_auto | 6.88 | 0.032* | 0.032 * |

**Table S9: Mann Whitney U test results for Study Area Pairwise Comparisons vs Performance (Recall, Precision, F1 Score, F2 Score, F3 Score)**

| Metric | Comparison | U Statistic | Raw p-value | Adjusted p-value |
| --- | --- | --- | --- | --- |
| precision_auto | CEEDER vs Campbell | 78.0 | 0.008* | 0.024* |
| precision_auto | CEEDER vs Cochrane | 35.0 | 0.696 | 1.000 |
| precision_auto | Campbell vs Cochrane | 18.0 | 0.093 | 0.278 |
| f1_score_auto | CEEDER vs Campbell | 78.0 | 0.008* | 0.024* |
| f1_score_auto | CEEDER vs Cochrane | 39.0 | 0.965 | 1.000 |
| f1_score_auto | Campbell vs Cochrane | 20.0 | 0.139 | 0.416 |
| f2_score_auto | CEEDER vs Campbell | 78.0 | 0.008* | 0.024* |
| f2_score_auto | CEEDER vs Cochrane | 36.0 | 0.762 | 1.000 |
| f2_score_auto | Campbell vs Cochrane | 18.0 | 0.093 | 0.278 |
| f3_score_auto | CEEDER vs Campbell | 78.0 | 0.008* | 0.024* |
| f3_score_auto | CEEDER vs Cochrane | 36.0 | 0.762 | 1.000 |
| f3_score_auto | Campbell vs Cochrane | 18.0 | 0.093 | 0.278 |

**Table S10: Kruskal Wallis test results for Seed Article Type vs Performance (Recall, Precision, F1 Score, F2 Score, F3 Score)**

| Metric | Kruskal Statistic | Kruskal p-value (Raw) | Kruskal p-value (Adjusted) |
| --- | --- | --- | --- |
| recall_auto | 6.96 | 0.324 | 0.324 |
| precision_auto | 4.75 | 0.577 | 0.577 |
| f1_score_auto | 2.56 | 0.861 | 0.861 |
| f2_score_auto | 4.10 | 0.663 | 0.663 |
| f3_score_auto | 4.42 | 0.620 | 0.620 |

**Table S11: Kruskal Wallis test results for Inclusion Criteria Type vs Performance (Recall, Precision, F1 Score, F2 Score, F3 Score)**

| Metric | Kruskal Statistic | Kruskal p-value (Raw) | Kruskal p-value (Adjusted) |
| --- | --- | --- | --- |
| recall_auto | 0.01 | 0.912 | 0.912 |
| precision_auto | 1.22 | 0.268 | 0.268 |
| f1_score_auto | 0.78 | 0.376 | 0.376 |
| f2_score_auto | 1.11 | 0.293 | 0.293 |
| f3_score_auto | 1.11 | 0.293 | 0.293 |

**Table S12: Kruskal Wallis test results for API Choice vs Performance (Recall, Precision, F1 Score, F2 Score, F3 Score)**

| Metric | Comparison | U Statistic | Raw p-value | Adjusted p-value | Significant |
| --- | --- | --- | --- | --- | --- |
| recall_auto | openalex vs semanticscholar | 326.50 | 0.516 | 0.516 |  |
| precision_auto | openalex vs semanticscholar | 375.00 | 0.863 | 0.863 |  |
| f1_score_auto | openalex vs semanticscholar | 362.00 | 0.972 | 0.972 |  |
| f2_score_auto | openalex vs semanticscholar | 371.00 | 0.917 | 0.917 |  |
| f3_score_auto | openalex vs semanticscholar | 372.00 | 0.904 | 0.904 |  |
